# Rightward Tympanic Membrane Temperature Bias During Acute Restraint-Isolation Stress in Marmoset Monkeys

**DOI:** 10.3389/fnins.2019.00913

**Published:** 2019-08-30

**Authors:** Lucas C. Pereira, Rafael S. Maior, Marilia Barros

**Affiliations:** ^1^Primate Center, Institute of Biology, University of Brasília, Brasília, Brazil; ^2^Department of Pharmacy, School of Health Sciences, University of Brasília, Brasília, Brazil; ^3^Department of Physiological Sciences, Institute of Biology, University of Brasília, Brasília, Brazil

**Keywords:** restraint, isolation, cortisol, tympanic membrane temperature, hemisphere asymmetry, marmoset

## Abstract

Restraint is widely used to experimentally assess stress-induced effects. Surprisingly, little is known on how marmosets – an increasingly used small primate – process and respond to restraint stress. Here, we assessed blood cortisol concentration and tympanic membrane temperatures (TMT) in adult marmoset monkeys (*Callithrix penicillata*) during 0, 15, or 30 min of restraint and social isolation in a small cage. TMT reflects blood flow to the cerebral hemispheres, which in turn reflects neural activity. Baseline TMT were subtracted from post-test measures to establish shifts in blood flow possibly induced by ipsilateral brain activity. Cortisol was assayed immediately after the post-test assessment of the TMT. Marmosets restrained-isolated for 15 or 30 min had higher cortisol levels than the non-restrained-isolated group. Furthermore, significant changes in TMT were detected only in the right ear of the restrained-isolated groups, this effect being unrelated to overall body temperature or the time needed to capture/measure the TMT. Adult marmosets thus readily perceive a significant reduction in their range of movement as an event of sufficient negative intensity and/or duration to activate a pertinent neuroendocrine response. Also, an asymmetrical shift in their TMT reflects that such an aversive event may be rightwardly biased in this primate.

## Introduction

Acute restraint is a widely adopted means to experimentally assess the effects of stress-related events in animals. It is a simple, low-cost, painless and reversible procedure that involves restricting limb movement or significantly limiting the range of motion (Buynitsky and Mostofsky, 2009). This inherently induces psychological distress, which potentially causes adverse physiological and behavioral effects, such as hypothalamus-pituitary-adrenal (HPA) axis hyperactivity, immunosuppression, impaired memory and decreased motivation (Glavin et al., 1994; Buynitsky and Mostofsky, 2009). It should be noted that recurring restraint-isolation events are also required for the management of captive animals.

Marmosets are a family of diurnal and arboreal neotropical non-human primate (NHP). These small-bodied animals are being increasingly adopted as a translational model of several human diseases (‘t Hart et al., 2012) and consequently the number of laboratory-based colonies has increased. To generate more replicable high-quality data in such prominent animal models, captivity-induced stress needs to be properly addressed and significantly attenuated (Rennie and Buchanan-Smith, 2006). Therefore, different sources of husbandry-related stress are currently under investigation (e.g., *loud noises*: Kaplan et al., 2012; *social isolation*: Taylor et al., 2014; *human interaction*: Pereira et al., 2018), including restraint stress. This can be induced by brief manual immobilization for injection/sampling (Schultz-Darken, 2003) or lengthier sojourn in a confinement device (Schultz-Darken et al., 2004). In marmosets, such events activate the HPA-mediated stress response (i.e., cortisol; *Callithrix jacchu*s (common marmoset): Saltzman and Abbott, 2011; Aubert et al., 2013; *C. kuhlii*: Smith and French, 1997), as well as decrease luteinizing hormone levels (O’Byrne et al., 1988), sperm count (Cui, 1996) and sexual receptivity in common marmosets (Aubert et al., 2013).

The different behavioral and hormonal responses to acute restraint stress require complex and highly coordinated neural networks (Van de Kar et al., 1991; Murison and Overmier, 1993), which can be aided by hemisphere specialization. An asymmetrical brain is thought to have higher processing capacity, speed and efficiency (Vallortigara and Rogers, 2005). Although the specific hemisphere for processing emotion-laden stimuli is still under debate (reviewed in Gainotti, 2012), studies in marmosets (*C. jacchus*: Hook-Costigan and Rogers, 1998; Cameron and Rogers, 1999; Gordon and Rogers, 2015; *C. geoffroyi*: Braccini and Caine, 2009; *C. penicillata*: Souza Silva et al., 2007; Pereira et al., 2018), other animals and humans (reviewed in Rogers, 2009; Gainotti, 2012) have consistently found that detecting/responding to (negative) emotional stimuli is a rightwardly biased neural process. Hemisphere asymmetry is established by genetic/epigenetic factors interacting with environmental cues and subjective experiences (Rogers, 2014). The latter has important implications, given that prior experience can alter biased hemispheric control and influence long-term behavioral reactivity and stress-coping strategies (*common marmosets*: Gordon and Rogers, 2015; *rhesus monkeys*: Bethell et al., 2012). Tomaz et al. (2003) reported that, in marmosets (*C. penicillata*), the animals’ capture/restraint history predicted the extent of right-sided activity during a new episode. As the latter was assessed only once and immediately after the event, revealing no left-right asymmetry, the effect of different restraint stress intervals on the marmosets’ hemisphere activity requires further investigation.

Therefore, we assessed the tympanic membrane temperature (TMT) of captive adult black tufted-ear marmosets (*C. penicillata*) during a 15 and 30 min period of acute restraint in a small cage and in social isolation. TMT is a fast, inexpensive and non-invasive indirect index of real-time changes in hemisphere activity (Cherbuin and Brinkman, 2007; Propper and Brunyé, 2013), having already been used in this same species (Tomaz et al., 2003; Pereira et al., 2018). TMT reflects, via changes in blood flow, ipsilateral activity-induced changes in brain temperature (Baker et al., 1972; Schuman et al., 1999; Yablonskiy et al., 2000). We hypothesized that the marmosets’ right TMT would be altered by such an aversive event, particularly at the longer interval, yet we made no prediction for the directionality of the TMT shift. TMT does seem to reflect neural activity at the hemisphere level (Schiffer et al., 1999), yet the meaning of directional shifts in this relationship remains unresolved (i.e., increase vs. decrease TMT/activity; Propper and Brunyé, 2013). This can be attributed to considerable between-study differences in species, subject population (infant vs. adult), comparison mode (within vs. between-subject analysis), response measure (trait vs. state response), task motivation (approach vs. avoidance) and neural requirement (deep vs. superficial structures), in addition to variations in baseline body/brain temperatures and body mass (Sukstanskii and Yablonskiy, 2006; Propper and Brunyé, 2013). We also assessed blood cortisol concentration as an established hormonal measure of the neuroendocrine stress response.

## Materials and Methods

### Ethics Statement

The procedures herein were approved by the Animal Ethics Committee of the University of Brasilia (no. 006/2017) and carried out in accordance with the Brazilian regulations for the scientific use of laboratory animals (Lei Arouca 11.794/2008), as well as the CONCEA/Brazil and NIH/USA guidelines for the care and use of laboratory animals.

### Subjects and Housing Conditions

Fifteen adult black tufted-ear marmosets (*C. penicillata*; 7 males, 8 females) were used (mean age: 6.5 years old, range: 4.5–8.0 years old). They weighed 357 ± 10 g (mean ± sem; range: 310–435 g) at the beginning of the study and none were currently pregnant or recently had infants. The subjects were housed at the Primate Center of the University of Brasilia in heterosexual pairs, the exception being one female which was housed with her pairmate and a younger male sibling. Housing was held under natural light, temperature and humidity conditions in standard home-cages of a same colony room. This was a partially covered outdoor facility with two parallel rows of 12 home-cages each (2 × 1 × 2 m; W × L × H). Personnel and researchers could access the home-cages via a wire-mesh enclosed corridor located between the two rows of cages. Marmosets could not enter this corridor. A roof covered this central corridor and two-thirds of the length of each home-cage. Therefore, the marmosets had unrestrained access to both an uncovered area, as well as a shaded/protected area. The home-cages were provisioned with a nest-box, ropes, wood perches, a PVC tube for dry chow and a food tray for fresh items. The latter consisted of a mixture of pieces of fresh fruits and vegetables, along with boiled eggs, nuts, live mealworms and/or cooked chicken breast. These were provided daily at 07:00 h and unconsumed items were removed at 17:00 h. Water and chow were available *ad libitum*. The housing and maintenance conditions complied with the regulations of the Brazilian Institute of Environment and Renewable Natural Resources (IBAMA).

The subjects were all captive-born either at the Primate Center or transferred to this location from other facilities in Brazil at least six years prior to this study. They had also been submitted to routine veterinary procedures requiring capture and variable restraint intervals. We were thus unable to ascertain the subjects’ exact history with restraint procedures, yet they have been restrained >50 times in a manner similar to that used in present study. Furthermore, all subjects were implanted with a subcutaneous radio-frequency transponding identification microchip (BioThermo 985 LifeChip, Destron Fearing, South St. Paul, MN, United States) that provides a reliable subcutaneous temperature (SCT) measure within a range of 0 to 50°C (for more details see Pereira and Barros, 2016).

### TMT and SCT Assessment and Analyses

The marmosets’ right and left TMT were assessed with an infrared digital ear thermometer (IFR 100 Dual Mode Thermometer, Microlife, Brazil). It has an operating temperature range of 10 to 50°C, a sensitivity of 0.1°C and an accuracy of ±0.2°C (between 32.0 and 42.2°C), and can reliably assess the TMT of small neotropical primates (Pereira and Barros, 2016; Pereira et al., 2018). Six TMT readings were made, at 5 s intervals: three in the left ear and three in the right ear. These six measurements were taken one at a time, always alternating the side between each assessment. The first ear to be measured was determined arbitrarily. For each reading, the marmoset’s external ear was gently pulled up and back and the thermometer inserted into its right or left ear. The device was immediately activated and the temperature was displayed ∼1 s later. Of the three readings made in each ear, only the highest recorded temperature was used. This was done to minimize a possible error when positioning the thermometer, as the tympanic membrane is hotter than the surrounding tissue (Heusch et al., 2006).

Each subject’s SCT was also assessed to establish whether the possible changes in TMT were not related to a general effect on body temperature. For this we used a portable universal microchip reader (HS9002B Pocket Reader, Destron Fearing, São Caetano do Sul, Brazil; see section “Subjects and Housing Conditions”). When activated at ∼2 cm from the implant site, it provided the subjects’ SCT.

A SCT and TMT reading was performed immediately before and after the restraint-isolation test described below (section “Restraint-Isolation Stress Procedure”). The same person performed all measurements, using disposable probe covers in the case of the TMT thermometry. To account for individual variations in initial TMT and SCT, the pre-test measurement was subtracted from its respective post-test value, thus providing a difference score; i.e.: Δ = post-test temperature – pre-test temperature (in °C). A positive Δ value indicates that the temperature increased during the test trial, while a negative Δ score reveals a decrease in this measure.

### Restraint-Isolation Stress Procedure

The marmosets were randomly assigned to one of three experimental groups, each being submitted to a different acute restraint-isolation stress interval: 0, 15, or 30 min (groups RS-0 with 3 males and 2 females, RS-15 with 2 males and 3 females, and RS-30 with 2 males and 3 females, respectively). The procedure was held between 14:00 and 16:30 h, when the marmosets’ general body temperature and activity pattern remain constant (Hetherington, 1978; Hoffmann et al., 2012).

Each subject was tested only once, regardless of its group. This trial was divided into three consecutive steps: (1) a pre-test body temperature measurement, (2) a period of restraint-isolation, and (3) a post-test body temperature measurement. Behavioral measures were not recorded. The subject was quickly captured in the home-cage, manually restrained and its pre-test SCT and TMT were immediately assessed as described above (Step 1). The marmoset was subsequently placed in a familiar restraint cage (35 × 20 × 23; W × L × H in cm). The time required to capture the subjects and to assess their pre-test body temperatures was recorded (Table 1). For the RS-15 and RS-30 groups, the restraint cage was taken to a separate room located ∼50 m from the colony facility. Thus, for the duration of the pre-established restraint-isolation interval, the marmoset had no visual or olfactory contact with other colony members (Step 2). At the end of this interval, the subject was taken back to the colony room where it was removed from the cage and its post-test SCT and TMT were immediately assessed as already described (Step 3). For the control RS-0 group, the marmoset was immediately removed from the cage and the same two post-test body temperature measures were recorded, thus essentially omitting only Step 2 of the procedure held with the other two groups. The time required to obtain the subjects’ post-test body temperatures was also recorded (Table 1). Thereafter, the marmoset was taken to a procedure room adjacent to the colony facility, where it was submitted to the blood sampling procedure described below.

**TABLE 1:** Time required to perform different steps of the experimental procedure in the marmoset monkeys submitted to 0, 15, or 30 min interval of restraint-isolation stress (mean ± sem).

**Parameter**	**Restraint-isolation stress**		
	**00-min**	**15-min**	**30-min**
Pre-test home-cage capture (s)	19 ± 7	12 ± 3	26 ± 7
Pre-test temperature assessment (s)	50 ± 2	49 ± 1	49 ± 1
Post-test temperature assessment (s)	51 ± 2	50 ± 2	48 ± 1
Post-test blood sampling (min)	3.0 ± 0.4	2.5 ± 0.3	3.6 ± 0.4

### Blood Sampling and Cortisol Assay

In the presence of the veterinarian, a single blood sample was obtained from each subject following the post-test TMT assessment. For this, the animal was anesthetized by inhalation of isoflurane using a portable universal vaporizer kit (Brasmed Vetcase, São Paulo, Brazil) set at 2% and an oxygen flow of 1 L/min. A 0.5 mL blood sample was then obtained via femoral venipuncture, which was transferred to a 4 mL chilled vial containing clot activator and serum separator barrier gel (Vacuette, Brazil). After recovery (1–2 min), the subject was taken back to its home-cage and monitored for 15–30 min. The duration of the procedure was recorded to assess its possible influence on the cortisol content (Table 1).

Each blood sample was centrifuged for 5 min at 3.000 rpm and room temperature, and the serum transferred to a 0.5 mL polypropylene vial. The serum was then analyzed for cortisol content by single direct chemiluminescence immunoassay (CLIA) using a commercial kit for the automated ADVIA Centaur^®^ XP system (Siemens, Brazil) and a dilution of 1:50 (serum:diluent; Multi-diluent 3, Siemens, Brazil). The cortisol assay and dilution factor were based on a previous study in this species using the same blood sampling and analysis procedure (Pereira et al., 2018). Cortisol assay sensitivity was 1 μg/dL, and inter- and intra-assay coefficient of variation from pooled serum were 9.8% and 7.5%, respectively.

### Statistical Analyses

Data were found to be normally distributed and with equal variance, as assessed via Shapiro-Wilk and Levene’s test, respectively, and thus analyzed using parametric statistics on raw non-transformed values. Data on TMT Δ scores were analyzed to establish possible differences between the left and right side and the restraint-isolation intervals. For this, a mixed-design two-way analysis of variance (ANOVA) was used, with SCT Δ scores as a covariate, as well as “interval” as the independent factor (0 × 15 × 30 min) and “side” as the repeated measure variable (left × right). Data on cortisol, initial body mass and SCT Δ scores were assessed for between-group effects via one-way ANOVA. Whenever significant effects were obtained in the ANOVA analyses, subsequent comparisons were performed using Tukey’s test. In addition, Pearson’s correlation test was used to established the relationship between: (1) TMT Δ scores and cortisol content; (2) TMT Δ scores and the respective capture/temperature assessment time; (3) pre-test TMT and home-cage capture time; (4) TMT and SCT Δ scores; and (5) cortisol concentrations and the corresponding time required to obtain the blood sample. Significance level for all tests was set at *p* ≤ 0.05.

## Results

**FIGURE 1:**
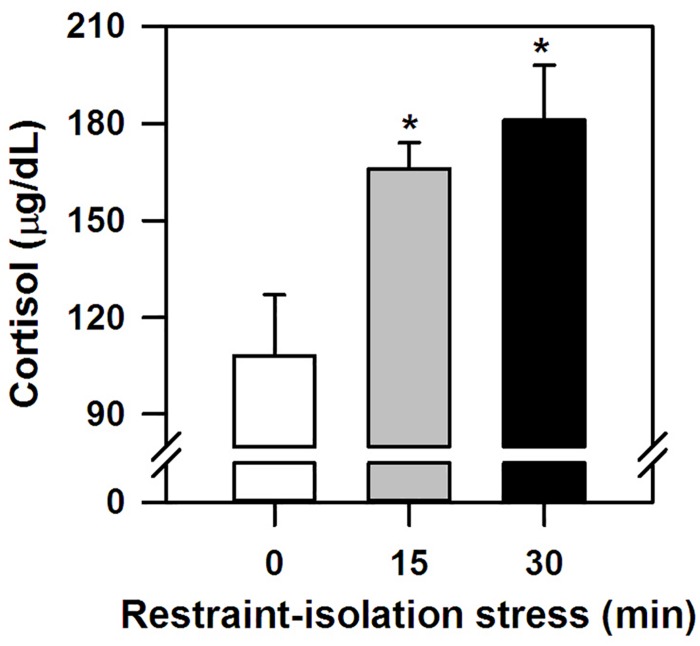
Cortisol concentration (mean + sem) detected in the marmosets immediately after 0-, 15- or 30-min period of restraint-isolation stress. *n* = 5/group; ^∗^*p* < 0.05 15- and 30-min groups vs. 0-min control group.

The marmosets submitted to the (15 and 30 min) period of restraint-isolation had similar cortisol concentrations, which in turn were significantly higher than those of the RS-0 control group (*F*_2__,__14_ = 5.61, *p* = 0.02; Figure 1). Importantly, cortisol values were not correlated with the time required to obtain the blood sample (Table 1; RS-0: *r* = −0.06, *p* = 0.92; RS-15: *r* = −0.10, *p* = 0.88; RS-30: *r* = −0.39, *p* = 0.52).

**FIGURE 2:**
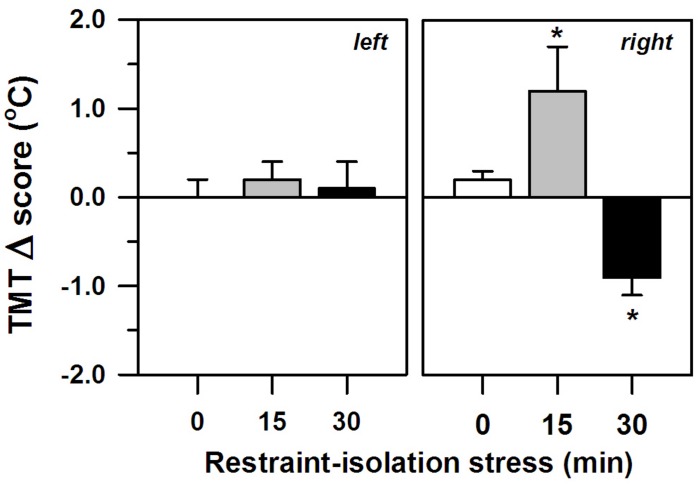
Change in the marmosets’ left and right tympanic membrane temperatures (TMT, Δ score; mean + sem) in response to 0-, 15- or 30-min period of restraint-isolation stress. TMT Δ score = post-test temperature – pre-test temperature in °C; *n* = 5/group; ^∗^*p* < 0.05 vs. 0-min interval (right TMT).

The two periods of restraint-isolation also significantly altered the marmosets’ TMT, albeit only in their right ear (interval effect: *F*_2__,__11_ = 5.34, *p* = 0.02; side effect: *F*_1__,__11_ = 0.20, *p* = 0.66; interaction: *F*_2__,__11_ = 5.56, *p* = 0.02; Figure 2), with no significant effect of the SCT Δ scores (*F*_1__,__11_ = 0.10, *p* = 0.76). The right TMT increased significantly in the RS-15 group, whereas it decreased in the RS-30 group. This differed from the RS-0 control group whose right TMT remained unaltered. The left TMT also remained constant, regardless of the restraint-isolation interval. Cortisol content was not associated with the left (RS-0: *r* = −0.76, *p* = 0.13; RS-15: *r* = 0.41, *p* = 0.49; RS-30: *r* = 0.10, *p* = 0.88) or the right TMT Δ scores (RS-0: *r* = −0.71, *p* = 0.18; RS-15: *r* = −0.25, *p* = 0.69; RS-30: *r* = −0.08, *p* = 0.90).

Importantly, both the pre-test TMT (*capture time*: x left TMT – *r* = 0.38, *p* = 0.14; x right TMT – *r* = 0.19, *p* = 0.49) and the general TMT Δ scores were not associated with the time required to initially capture the marmosets in their home-cages (Table 1; *capture time*: x left TMT Δ score – *r* = −0.41, *p* = 0.13; x right TMT Δ score – *r* = −0.48, *p* = 0.08). TMT Δ scores were not associated with the time needed to assess the body temperatures pre- or post-test (Table 1; *left TMT*Δ *score*: x pre-test assessment – *r* = 0.21, *p* = 0.46; x post-test assessment: *r* = 0.13, *p* = 0.64; *right TMT*Δ *score*: x pre-test assessment – *r* = 0.70, *p* = 0.81; x post-test assessment: *r* = 0.11, *r* = 0.20). The change observed in the TMT was also not correlated with that of the SCT (Table 2; *SCT*Δ *score x TMT*Δ *score:* left side – *r* = 0.35, *p* = 0.20; right side – *r* = 0.29, *p* = 0.30). SCT Δ scores (*F*_2__,__14_ = 0.81, *p* = 0.47) and body mass did not vary between groups (*F*_2__,__14_ = 1.29, *p* = 0.31; Table 2).

**TABLE 2:** Body mass and change in subcutaneous temperature (SCT Δ) of marmoset monkeys submitted to 0, 15, or 30 min interval of restraint-isolation stress (mean ± sem).

**Parameter**	**Restraint-isolation stress**		
	**00-min**	**15-min**	**30-min**
body mass (g)	372 ± 20	334 ± 12	364 ± 12
SCT Δ score (°C)^a^	−0.2 ± 0.2	0.1 ± 0.1	0.0 ± 0.2

*^*a*^Δ score = post-trial temperature – pre-trial temperature.*

## Discussion

Here we demonstrated that when marmoset monkeys were restrained and isolated for a single short period of time in a small cage (groups RS-15 and RS-30), their circulating cortisol levels were significantly higher than in non-restrained-isolated individuals (control group RS-0). In this latter group, blood cortisol concentrations were comparable to those of non-stressed monkeys in previous studies (*C. penicillata*: Lima et al., 2008; *C. jacchus*: Pryce et al., 2002; Saltzman and Abbott, 2011). Also, hormone levels were not related to the time required to obtain the blood samples, this being done in <5min (Saltzman et al., 1994). Both short (15–50 min) and more prolonged (11 h) periods of restraint can markedly increase cortisol concentrations in marmosets (*C. jacchus*: Saltzman and Abbott, 2011; Aubert et al., 2013; *C. kuhlii*: Smith and French, 1997). In other NHP, cortisol levels also rose due to an acute restraint period *per se* (e.g., *capuchin*: Lahoz et al., 2007; *rhesus*: Morrow-Tesch et al., 1993; *spider monkey*: Rodas-Martínez et al., 2013) or simply by observing other monkeys being restrained (Gilbert and Baker, 2011). Increases in HPA activity have been reported in rodents as well (reviewed in Buynitsky and Mostofsky, 2009). On the other hand, recurrent episodes may lead to progressively blunted HPA reactivity as a means to physiologically adapt to sustained high glucocorticoid concentrations (Ruys et al., 2004; Paramastri et al., 2007). Acute restraint-induced cortisol release can be influenced by stress intensity/duration, age and trait-like characteristics (reviewed in Buynitsky and Mostofsky, 2009). Furthermore, male marmosets may have a higher cortisol response to capture/restraint-isolation than females (*C. kuhlii*: Smith and French, 1997), with sex differences also being reported for predatory stress events (*C. jacchus*: Cross and Rogers, 2006; Saltzman and Abbott, 2011; *C. penicillata*: Pereira et al., 2018). Due to the variable and small sample size for each sex in the present study (2–3 individuals), a gender analysis was not conducted. Although we were also unable to ascertain our subjects’ prior restraint history, this is not a predictable or systematically held procedure at our Primate Center. As argued by Dallman (2007), the likelihood of habituating to restraint is significantly influenced by exposure frequency. Accordingly, our adult marmosets seemed to perceive even a short period of restraint-isolation as a stressful event of sufficient negative intensity and/or duration to activate a pertinent neuroendocrine response.

Notably, we also detected significant shifts in the right TMT of the restrained-isolated animals – an effect not seen in the left ear or the RS-0 control group. This was not related to overall body temperature (i.e., SCT), which remained unaltered, or the time needed to capture the animal or to obtain the temperature readings. Moreover, the different groups did not differ in terms of body mass. A change in TMT may reflect, however, an ipsilateral shift in brain temperature and blood flow (Baker et al., 1972; Schuman et al., 1999; Yablonskiy et al., 2000) that derives from neuronal activity (Schiffer et al., 1999) and thereby is viewed as an indirect index of real-time changes in hemispheric function (Cherbuin and Brinkman, 2007; Propper and Brunyé, 2013). In fact, neuroimaging studies have detected an increase in blood flow only to the right hemisphere when humans view emotionally negative stimuli (Canli et al., 1998). In this perspective, our short-term events may have been asymmetrically processed by the marmosets’ right hemisphere. Restraint-initiated stress does seem to require complex neural processing (Van de Kar et al., 1991; Murison and Overmier, 1993). While Tomaz et al. (2003) reported similar findings using this same measure and species, Hanbury et al. (2011) detected a bilateral increase in TMT as a result of a brief (6 min) restraint event in bushbabies. Stress duration and/or species variability may possibly account for this apparent discrepancy requiring further investigation.

As seen in our study, central mechanisms for processing fearfulness and negative affect do seem to be rightwardly biased in both humans and several animals (reviewed in Rogers, 2009; Gainotti, 2012). This was the case for marmosets during a brief capture/restraint procedure (*C. penicillata*: Tomaz et al., 2003), exposure to novelty (*C. jacchus*: Cameron and Rogers, 1999) and confrontation with predator-related stimuli (*C. jacchus*: Hook-Costigan and Rogers, 1998 (*C. penicillata*: Souza Silva et al., 2007; Pereira et al., 2018). In other NHP, the right hemisphere also preferentially processed negative socially relevant events (e.g., *chimpanzees*: Parr and Hopkins, 2000; *rhesus*: Kalin et al., 1998; *baboons*: Wallez and Vauclair, 2011). This lateralization of emotional processing seems to lead to faster and longer lasting behavioral and physiological responses (Hauser, 1993; Wittling and Roschmann, 1993; Kalin et al., 1998; Parr and Hopkins, 2000). It should be noted, though, that we used a small sample size and did not assess stimuli with different valence (negative, positive and neutral). Therefore, our results should not be taken as explicit evidence for any of the conceptual models of hemispheric specialization for emotionality (i.e., valence vs. right hemisphere hypotheses; reviewed in Gainotti, 2012).

The specific direction in which the right TMT shifted in the present study depended on the duration of the restraint-isolation interval – it increased after 15 min, yet decreased after 30 min. An increase in TMT has been associated with an ipsilateral increase in hemispheric activity due to emotional stress in other between-subject comparison studies in NHP (*bushbabies*: Hanbury et al., 2011, 2013; *chimpanzees*: Parr and Hopkins, 2000) and humans (e.g., Propper and Brunyé, 2013). However, lower brain temperatures were recorded after direct limbic stimulation in *rhesus* monkeys (Hayward and Baker, 1968) and predation stress decreased TMT in C. *penicillata* (Pereira et al., 2018). The exact mechanisms for how local temperatures change during brain activity and how TMT relates to hemispheric activity are still unresolved. Several aspects may account for the current directional disparity in which the TMT shifted. Brain temperature and heat dissipation mechanisms differ between superficial and deep areas (Sukstanskii and Yablonskiy, 2006). As such, different structures may have been recruited at different time lags, even within the same hemisphere. Alternatively, some brain regions can be temporarily deactivated, leading to lower blood flow (Raichle et al., 2001). Concomitant lateralization of cognition function could have also affected cerebral and TMT temperatures. In chimpanzees (Hopkins and Fowler, 1998) and humans (Meiners and Dabbs, 1977; however, see Cherbuin and Brinkman, 2007) the right TMT decreases task-dependently. It should be noted, though, that autonomic thermoregulatory processes mediate brain temperature and blood flow (e.g., Hayward and Baker, 1968). Aspects unrelated to neural activity could have thus contributed to the current directional shifts in TMT, particularly at the 30 min interval. In addition, although negative stimuli can lead to long-lasting responses, it has been argued that TMT only reliably predicts changes in hemispheric activity for up to 10–20 min (Parr and Hopkins, 2000; Cherbuin and Brinkman, 2007), while neuroimaging studies also based on shifts in blood flow indicate possibly shorter intervals (e.g., Hervé et al., 2013). Thus caution should be taken when interpreting the results in the RS-30 group. Whether TMT equally reflects heat dissipation and local change in blood flow induced by neuronal activity, as well as their specific temporal profiles, remains to be assessed. Nonetheless, as subjects were tested during a period that does not correspond to their bimodal peak in foraging behavior (Nunes et al., 2010), a lack of energy/metabolism is probably not responsible for the TMT decrease in the RS-30 group. Taken together, we are still uncertain as to the functional significance of the specific and opposing shifts in TMT.

In summary, our results indicate that adult marmoset monkeys readily perceive being acutely restrained as a valid threat source and thereby activate well-established neuroendocrine stress responses (i.e., cortisol). The asymmetrical shift in TMT seems to reflect that such an aversive event may be rightwardly biased in this primate. As we were unable to ascertain prior restraint history or establish possible sex differences due to the variable and small number of males/females, caution should be taken when generalizing these results. Sex differences in stress-induced cortisol release (e.g., Pereira et al., 2018) and general hemisphere asymmetry are reported (reviewed in Rogers, 2014). Gonadal hormones may act during developmental stages and/or modulate adult neural circuits, as well as prompt indirect influences (e.g., sex-specific behaviors and/or cognitive abilities; Rogers, 2014). Further studies are thus required. Our results also indicate that TMT thermometry acted as an inexpensive, fast, non-invasive and indirect index of real-time hemisphere activity in this small primate. Our results also provide important insights for the welfare and captive management of this small neotropical primate, particularly when considering the use of cage-restraint for clinical and/or experimental purposes. How cortisol and TMT measures respond to repeated exposures, and how specific directional shifts in TMT relate to cerebral hemisphere activity during aversive events require further investigation.

## Data Availability

All datasets generated for this study are included in the manuscript and/or the supplementary files.

## Ethics Statement

This study was carried out in accordance with the Brazilian regulations for the scientific use of laboratory animals (Lei Arouca 11.794/2008), as well as the CONCEA/Brazil and NIH/USA guidelines for the care and use of laboratory animals. The protocol was approved by the Animal Ethics Committee of the University of Brasilia (No. 006/2017).

## Author Contributions

LP and MB conceived and designed the study, acquired, analyzed, and interpreted the data, and drafted and critically revised the manuscript for important intellectual content. RM analyzed and interpreted the data, and drafted and critically revised the manuscript for important intellectual content.

## Conflict of Interest Statement

The authors declare that the research was conducted in the absence of any commercial or financial relationships that could be construed as a potential conflict of interest.
